# Differential Probability Discounting Rates of Gamblers in an American Indian Population

**DOI:** 10.3389/fnbeh.2022.809963

**Published:** 2022-02-16

**Authors:** Tadd D. Schneider, Jordyn A. Gunville, Vlad B. Papa, Morgan G. Brucks, Christine M. Daley, Laura E. Martin, David P. Jarmolowicz

**Affiliations:** ^1^Department of Applied Behavioral Science, University of Kansas, Lawrence, KS, United States; ^2^Cofrin Logan Center for Addiction Research and Treatment, University of Kansas, Lawrence, KS, United States; ^3^Center for American Indian Community Health, University of Kansas Medical Center, Kansas City, KS, United States; ^4^Hoglund Biomedical Imaging Center, University of Kansas Medical Center, Kansas City, KS, United States; ^5^Healthcare Institute for Improvements in Quality, University of Missouri-Kansas City, Kansas City, MO, United States

**Keywords:** probability discounting, gambling, American Indian, fMRI, behavioral economics

## Abstract

Probability discounting, a subset of behavioral economic research, has a rich history of investigating choice behavior, especially as it pertains to risky decision making. Gambling involves both choice behavior and risky decision making which makes it an ideal behavior to investigate with discounting tasks. With proximity to a casino being one of the biggest risk factors, studies into the American Indian population have been a neglected population of study. Using outcome measures from a pre-scan probability discounting task, the current study equated the scan task to evaluate behavioral and neurobiological differences in gamblers vs. non-gamblers. Gamblers showed differences in behavioral tasks (lower discounting rates) but not in patterns of neural activation.

## Introduction

In the United States, more than 80% of adults engage in some form of gambling each year (Barnes et al., 2017). This pattern is particularly pervasive amongst American Indians (AI). For example, in the past year, 76.9% of white Americans engaged in gambling, whereas 80.1% of AI gambled (Barnes et al., 2017). The discrepancies become even more pronounced as we consider those that frequently gamble and/or engage in problem gambling. Specifically, 9.3% of white Americans engaged in frequent gambling, with 1.8% reaching pathological criteria. By contrast, 12.6% of AI’s frequently gambled with 10.5% meeting pathological gambling criteria (Welte et al., 2001). Although gambling availability and types are constantly changing, high percentages of pathological gamblers (PG) engage in traditional casino games (22.5%), electronic gambling machines (18%), and numbers/lotto (5%; Binde et al., 2017).

One reason that PG risk may be elevated in AIs is that many live near casinos. Of the 562 AI tribes, The National Indian Gaming Commission estimates more than 240 tribes offer gambling activities at nearly 500 casinos (Ashton, 2002). Further, approximately half of AIs residing in the continental United States belong to tribes that operate a casino-style gaming operations on tribal lands (Evans and Topoleski, 2002). Of note, those who reside within 10 miles of a casino were twice as likely to have issues with problem gambling (Welte et al., 2004). In a study of 7th–12th grade AI children, approximately 75% had gambled in the last year (Peacock et al., 1999); much higher than the national average of 45–55% (Winters and Anderson, 2000; Stinchfield, 2011). Further, in a survey of public school students in Minnesota, 17.4% of the AI children reported daily/weekly gambling behavior, compared to 12.3% of the white children (Stinchfield et al., 1997). Although there are economic benefits to allowing casinos on their lands, it also brings a potential for unintended problems that put this population at risk.

Gambling often entails wagering a small amount of money for the chance to win a larger sum of cash. In behavioral economics, these sorts of tradeoffs are analoged via probability discounting tasks. Probability discounting (PD) tasks have subjects choose between smaller but guaranteed sums of money and larger yet uncertain sums of money. For example, a subject may choose between $50 and 95% chance of receiving $100. The presented options are typically titrated until the value of the two alternatives are subjectively equivalent (e.g., a subject may find a 95% chance of receiving $70 is as appealing as receiving $50). These points of subjective equivalence—called indifference points—are typically collected across a range of probabilities. By using Rachlin et al.’s (1991) hyperboloid equation to fit a function through those indifference points, the rate (*h*) at which the subject value (V) of some amount (A) the uncertain reward declines as rewards become less probable (represented as increasing odds against ([θ = (1-p)/p]; Rachlin et al., 1991) can be calculated using:


(1) $$V=A/\left(1+h\,\theta^{s}\right)$$

In doing so, *h* represents the speed at which V declines as uncertainty increases, frequently called the PD rate (Green et al., 1999; Estle et al., 2006). In simpler terms, smaller *h* values demonstrate a willingness to take risks, whereas larger values reflect aversiveness to risk (Peters and Buchel, 2009). Gamblers, who are more prone to risky behaviors (Hewig et al., 2010), demonstrate more shallow discounting across probabilities than controls (Holt et al., 2003; Madden et al., 2009; Miedl et al., 2012). Additionally, PD rates have a negative correlation with scores on the South Oaks Gambling Screener (Holt et al., 2003; Madden et al., 2009). These relations, however, have not been widely investigated in AIs (cf. Weatherly et al., 2012)—despite their elevated risk of PG. Specifically, although Weatherly et al. (2012) examined PD in AI’s the comparison between subjects suffering from GD and controls was not made.

Moreover, relatively little is known about the neurobiological processes driving PG. One approach to uncovering these important neuro-correlates is Functional Magnetic Resonance Imaging (fMRI). fMRI studies use Blood Oxygenation Level Dependence (BOLD) measures to evaluate changes in blood oxygenation levels during task involvement. Higher levels of activity require more oxygen, and therefore, require more blood flow for oxygenation. Measurements are collected while participants simultaneously complete a behavioral and/or neuropsychological tasks, such as simulated casino games (Miedl et al., 2010) or probability discounting (Peters and Buchel, 2009; Miedl et al., 2012).

Using probability discounting tasks in combination with fMRI, Peters and Buchel (2009) examined specific ROIs [ventral striatum (VS) and orbito-frontal cortex (OFC)] as participants completed discounting tasks. Using pre-scan indifference points from a probability discounting task, researchers equated the scan tasks so that each participant would make approximately 50% of choices for the smaller/certain and 50% for the larger/uncertain outcomes. This assured that there were enough trials wherein the subject chose each reward type (i.e., smaller certain, larger uncertain) to make valid comparisons. Significant results were seen in both the VS and OFC when subjects were coding for subjective value of the delayed or probabilistic rewards. Peters and Buchel (2009) noted that the VS and OFC are part of an integrated system that is activated when subjects are making decisions about rewards. Additionally, studies have found decreased activity in the VS and OFC when subjects were making decisions about delayed/probabilistic rewards during risky (low probability or long delay) reward trials (Miedl et al., 2012).

Studies examining the neuro-correlates of PD have added and will continue to add to our understanding of this behavioral process and its relation to PG. The extent which prior findings generalize to AIs—with their elevated risk of GD—remains unknown. The purpose of the current study was to examine PD and its neuro- correlates among AIs with and without PG—with the hope of extending the generality of prior findings.

## Materials and Methods

### Participants

American Indians (ages of 18–65) were recruited by the Center for American Indian Community Health (CAICH). Participants were 24 AIs of differing tribes spanning the Midwest plains. Using DSM-V criteria 12 gamblers and 12 controls were recruited with mean ages of 39 for gamblers (*SD* = 19.05) and 36 for controls (*SD* = 11.51). During recruitment, care was taken to ensure participants’ demographic characteristics were representative of the overall AI population. Participants were excluded from participation if they reported any condition contraindicating fMRI, current use of psychotropic medication, current or past abuse of illicit substances, diagnosis of severe neurological or psychiatric illness, inability to read and speak English fluently, left-handedness, or pregnancy. All participants were compensated $115 and a $20 gas card for their time in the study.

### Procedures

Upon arriving at Hoglund Biomedical Imaging Center at Kansas University Medical Center, participants were escorted to a consultation room. The consultation room was 8′× 12′ with a bank of windows along one wall. The other wall had a door and bookshelf. There was a round table with chairs in the middle of the room and a couch to the side. Written consent was obtained, then all other paperwork was completed, including demographics, payment form, and the MR safety screener. Participants then completed a PD task. Participants were then brought to a locker room and instructed to change into scrubs and remove any jewelry. Once changed into scrubs, participants were taken into the scanner. Participants requiring glasses were fitted with scanner compatible prescription goggles, and sight was checked by technician before their fMRI session.

### Probability Discounting Task (Pre-scan)

Participants completed a probability discounting task conducted on an encrypted laptop computer. In this task, participants were told,

*“Now, you’ll be making decisions about some probability of receiving some amount of money. You’ll see different probabilities of receiving amounts of money. Although you will not receive these amounts, pretend you will have the chance of receiving the amount and answer honestly. You can select between the two options by pressing the 1 and 2 buttons on this line of numbers. Press the 1 button for the option on the left and the 2-button for the option on the right.”*

Participants then completed four rounds of PD decision making, one round at each of the probabilities (90, 70, 50, and 10%). Probabilities were presented in descending order and all trials were completed for each probability before moving on to the next. On the first trial, participants are presented with a choice between a smaller, yet certain outcome (100% chance of $50), vs. a larger, probabilistic outcome (probabilistic chance of $100). If the participant chose the larger, uncertain reward, the value of the smaller, certain reward increased by 50% of the previous titration value (initially $25), but if the participant chose the smaller, certain reward, the value of the smaller, certain reward was reduced by 50% of the previous titration value. After the sixth titration at each probability the value of the smaller, certain reward was the participant’s indifference point. After completion of the task, research assistants retrieved the indifference points from the computer. These values were later entered into the task program in the scanner to equate the tasks for all participants.

### Functional Magnetic Resonance Imaging Scan

Scanning was performed on a 3-Tesla full body Siemens Skyra scanner (Siemens, Erlangen, Germany) fitted with a 20-channel head and neck coil.

Scans collected included an anatomical scan and three functional probability discounting task runs. T1-weighted 3D MPRAGE anatomic images were obtained (TR/TE 2,300/2.95 ms, flip angle 9°, FOV = 256 mm, matrix = 240 × 256, slice thickness = 1.2 mm). These images provided slice localization for functional scans and co-registration with fMRI data. Gradient echo blood oxygen level dependent (BOLD) scans were acquired in 43 interleaved slices at a 40° angle to the AC/PC line (TR/TE = 2,500/25.0 ms, flip angle = 90, matrix = 80 × 80, slice thickness = 3 mm, in-plane resolution = 2.9 mm). The duration of each functional run varied based on individual participant reaction times.

Anatomical scans were acquired for participant positioning. Indifference points from the practice rounds were entered for each participant to equate the difficulty of the task across participants. The task adjusted the dollar amounts presented at each probability to offer the same number of choices above and below pre-scan indifference points to each participant. The function of equating the tasks across participants was to prevent markedly different patterns of choice to more easily investigate the processes that support choice, rather than the choices that were made.

Participants were given a control pad with two buttons, side-by-side, that correlated with the choices projected onto the screen. The MR tech made sure the screen was visible by the participant and any last-minute adjustments were made. Instructions were given by the research assistant about the PD trials. Instructions were verbally delivered as before:

*“Now, you’ll be making decisions about some probability of receiving some amount of money. You’ll see different probabilities of receiving amounts of money. Although you will not receive these amounts, pretend you will have the chance of receiving the amount and answer honestly. You can select between the two options by pressing the left and right buttons on the controller. Press the left button for the option on the left and the right button for the option on the right.”*

Once instructions were delivered, the program was loaded and automatically triggered by the start of the scanner. All stimuli (PD choices) were presented using E-Prime (Psychology Software Tools, Inc., Sharpsburg, PA) for the scan portion of the task. The same adjusting amount PD procedure was used from the pre-scan testing, however, for the scan task, percentages were displayed in a pseudorandomized order. The screen above the participant showed the two options (the certain and probabilistic outcomes) the participant was to choose from. Options were presented in black text on a white background, with the certain outcome being randomized between the right and left side of the screen for each trial. Participants are presented with a choice between a smaller, yet certain outcome (100% chance of $50), vs. a larger, probabilistic outcome (probabilistic chance of $100). If the participant chose the larger, uncertain reward, the value of the smaller, certain reward increased by 50% of the previous titration value, but if the participant chose the smaller, certain reward, the value of the smaller, certain reward was reduced by 50% of the previous titration value. Participants made 32 choices per round, for three total rounds (total of 96 choices), to determine an indifference point at each probability. Between trials the instructions were repeated by the MR tech and each trial ended with a fixation cross that turned from black to gray to signify the end of the round.

After completing the scans, participants were escorted to a small office (5′× 7′) in which they completed additional questionnaires including timeline follow-back and SOGS questionnaire. Following completion of questionnaires, participants were escorted to the changing rooms to return to their street clothes. After changing, participants received their compensation and were thanked for their time.

## Analysis

### Behavioral Analysis

Probability discounting data were screened for orderliness using the criteria outlined by Johnson and Bickel (2008). Specifically, participants’ data were removed if an increase of more than 20% of the undiscounted amount was noted from one condition to the next, starting with the second indifference point, or if the final condition indifference point was not less than the first by at least 10%. Applying these criteria to the participant pool, three Gamblers and three Controls were removed for analyses of behavioral components.

Probability Discounting analyses and curve fitting were performed in GraphPad Prism (version 8), specifically Equation 1 (Rachlin’s Hyperboloid) was separately fit to the median indifference points for gamblers and controls using least squares regression. In doing so, the scaling parameter (s) was shared across groups, isolating the discounting rate (*h*) as the sole free parameter. Next, that shared scaling value (s) was input into the equation, and *h* values were calculated for each participant. These *h* values were used to examine correlations (Spearman) between discounting rates and SOGS scores. Additionally, PD rates were calculated using the AUC analysis. AUC is calculated using the trapezoid method that calculates the aggregate data (area) under the data path (curve) (Myerson et al., 2001). AUC provided a measure suitable for use with the parametric statistics used to examine between group differences in discounting rate.

### Functional Magnetic Resonance Imaging Analysis

All imaging data was collected and managed using RedCap electronic data capture tools hosted at University of Kansas Medical Center (Harris et al., 2009, 2019) for data quality checks. The quality of the fMRI data was checked for processing errors, alignment, and motion issues. Four subjects (two gambler and two control) were removed from imaging analysis due to not completing scans and two gamblers were removed due to excessive motion (i.e., > 50% censoring).

Data preprocessing and statistical analyses for imaging data were performed in AFNI (Cox, 1996). Preprocessing steps included motion correction, alignment, spatial smoothing and normalization. The fMRI images were realigned to the minimum outlier in each run to correct for motion. The images were spatially smoothed to 4 mm FWHM Gaussian kernel. Anatomic images were aligned to functional images and spatially normalized to Montreal Neurological Institute space using non-linear warping implemented with AFNI’s automated algorithm. Within each functional run were registered to the minimum outlier. Data points were censored if motion within a volume was greater than 0.3 mm. Statistical contrasts were conducted using multiple regression analysis with motion parameters included as nuisance regressors. Regressors representing the experimental conditions of interest (i.e., High, Mid, and Low Probability) were entered into the regression analysis using a duration modulated basis function. Timing files were created in Microsoft Excel to identify the beginning and end of each individual trial. Trials were separated into three groups (High, Mid and Low Probability). High probability trials consisted of the 90% probabilities, Mid probability trials consisted of the 70 and 50% probabilities, and the Low probability trials were set for the 10% probabilities. The quality of the fMRI data was checked for processing errors, alignment, and motion issues.

The data analysis focused on a whole-brain voxel-wise analysis of variance (ANOVA) implemented by AFNI’s 3 dMVM (Chen et al., 2014) to determine brain activation (i.e., percent signal change from baseline) main effects and interactions [Probability (Low, Mid, Hight) × Group (Gambler, Control). AFNI’s 3 dClustSim was used to estimate the probability of false positives and correct for multiple comparisons at *p* < 0.005 and α < 0.05.

## Results

**FIGURE 1:**
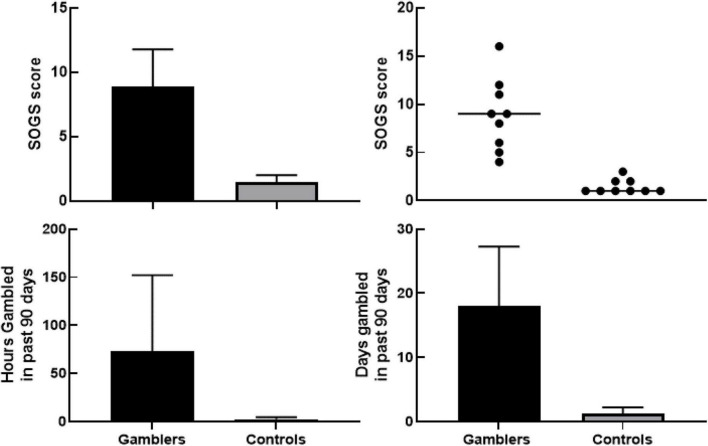
**Upper left panel** shows group differences in South Oaks Gambling Screener between gamblers and controls with 95% confidence interval using an independent samples *t*-test with Welch’s correction [*t*(16) = 5.837, *p* < 0.001]. **Upper right panel** shows scatterplot of individual South Oaks Gambling Screener values with the line representing median score per group. **Bottom left panel** shows group differences of number of hours gambled in the last 90 days with 95% confidence interval using a one-tailed independent samples *t*-test with Welch’s correction [*t*(8) = 2.034, *p* = 0.038]. **Bottom right panel** shows group differences in number of days gambled in last 90 days with 95% confidence interval using a one-tailed independent samples *t*-test with Welch’s correction [*t*(8) = 4.142, *p* < 0.002].

Figure 1 (top) shows South Oaks Gambling Scale scores for gamblers (range 4–16; *M* = 8.88. *SD* = 3.76) and controls (range 1–3; *M* = 1.44, *SD* = 0.73), with a significant difference between groups using an independent samples *t*-test [*t*(16) = 5.837, *p* < 0.001]. Figure 1 (bottom) shows participants’ histories of gambling involvement (hours and days). Results of previous studies have reported variance of gambling behaviors being unidirectional (gamblers). Our analytical hypothesis, therefore, was past gambling behavior variance would occur in one direction (gamblers). Using a one-tailed independent samples *t*-test with Welch’s correction resulted in a statistically significant [*t*(8) = 2.034, *p* = 0.038] difference in the number of hours gambled (Figure 1—bottom left) over the last 90 days between Gamblers (*M* = 65.73, *SD* = 94.02) and Controls (*M* = 2.00, *SD* = 2.68). Using the same analysis on self-reported days gambled in the last 90 days (Figure 1—bottom right) shows a statistically significant difference [*t*(8) = 4.142, *p* < 0.002] in the number of days gambled amongst Gamblers (*M* = 17.91, *SD* = 11.09) than Controls (*M* = 1.00, *SD* = 1.26).

**FIGURE 2:**
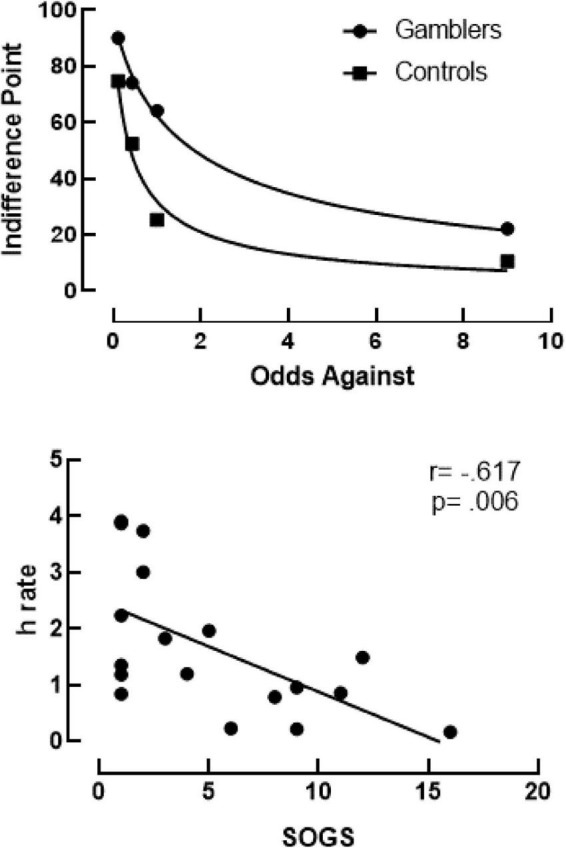
**Top panel** shows probability discounting curves using Rachlin’s Hyperboloid equation for gamblers (*R*^2^ = 0.9955) and controls (*R*^2^ = 0.9703). **Bottom panel** shows results of a Spearman correlation between discounting rates (*h*) on the y-axis and SOGS scores on the x-axis. The trendline shows a negative correlation of *r*(18) = -0.617, *p* = 0.006.

Figure 2 (top) shows the probability discounting curves fit to the median indifference points for PG (circles) and controls (squares) using Rachlin et al.’s (1991) hyperboloid discounting equation (Equation 1). This equation allows for two free parameters (discounting rate, *h*, and psychosocial scaling of delay, *s*) during analysis. To control for this, the scaling parameter (*s*) was held constant (i.e., shared) across all participants (*s* = 0.8165). Analysis showed an excellent fit for gamblers (*R*^2^ = 0.9955) and controls (*R*^2^ = 0.9703) to the group median. Additionally, discounting rates demonstrated a much more-shallow discounting rate by the gamblers (*h* = 0.6038) compared to controls (*h* = 2.134). When fitting Equation 1 to individual subjects’ data the group mean fit was fair for PG (*R*^2^ = 0.8642) and controls (*R*^2^ = 0.8926), with the mean log-transformed discounting rate (LN[*h*]) significantly differing between groups. As a confirmatory step, this analysis was also conducted using Area under the Curve. Area Under the Curve measures of indifference points were lower for Gamblers (*M* = 0.427, *SD* = 0.212) than Controls (*M* = 0.672, *SD* = 0.057). An unpaired *t*-test comparing AUC showed a statistically significant group difference [*t*(20) = –3.714, *p* ≤ 0.001]. Figure 2 (bottom) shows Spearman correlations between SOGS scores to discounting rates. Using a Spearman correlation analysis, results showed a significant negative correlation *r*(18) = -0.617, *p* = 0.006.

**FIGURE 3:**
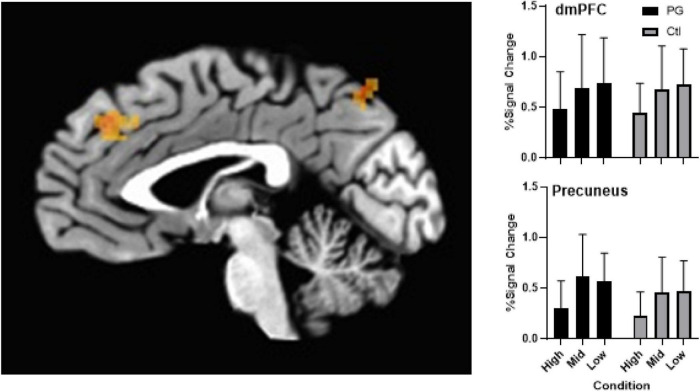
**Left panel** shows activation differences in the dmPFC and precuneus as an effect of condition (probability) of the probability discounting task during fMRI scan. **Upper right panel** shows group differences across probabilities in the dmPFC with a main effect of condition (error bars represent mean and SD). Controlling for multiple comparisons, results were significant at *p* < 0.005. **Bottom right panel** shows group differences across probabilities in the precuneus with a main effect of condition (error bars represent mean and SD). Controlling for multiple comparisons, results were significant at *p* < 0.005.

Whole brain analysis found no significant (*p* > 0.05) Group × Condition interaction or main effect of Group. A main effect of probability condition (Figure 3) was found in decision-making regions of the dorsal medial prefrontal cortex (dmPFC; x, y, z = -2, 44, 33, *p* < 0.005, corrected) and attention regions of the precuneus (x, y, z = -5, -69, 58), *p* < 0.005, corrected) demonstrating greater activation in low compared to high probability conditions.

## Discussion

Consistent with prior reports (Holt et al., 2003; Madden et al., 2009; Miedl et al., 2012) probability discounting rates were lower in PG relative to controls. Also consistent with prior studies, SOGS scores were negatively correlated with discounting rates (Holt et al., 2003; Madden et al., 2009). Also consistent with prior studies (Miedl et al., 2012), we did not obtain differences in task-related neural activation while PG and controls completed the PD task. There are four additional points we would like to make about these data.

First, despite the limited sample size, the between-group differences in probability discounting rate were robust. While this modest sample size is a limitation, the consistency of this finding with findings from prior studies (Holt et al., 2003; Madden et al., 2009) suggests that we were not capturing a spurious relation. As a systematic replication (Sidman, 1960) of prior studies in this novel and relevant population, the current findings strengthen our understanding of the relation between PD and GD. In light of prior findings, the current findings suggest probability discounting rates may be a behavioral process undergirding the risk taking seen in problem gambling. This possibility is strengthened by the replication of the negative relation between SOGS scores and PD seen in prior studies (Holt et al., 2003; Madden et al., 2009).

Second, the current study failed to find group-based differences in task-related neural activation when PG and controls completed the probability discounting task. This is consistent with prior studies (Peters and Buchel, 2009; Miedl et al., 2012), but may be based on the sample size providing insufficient power to demonstrate significant relations once corrected for multiple comparisons. Similar neurobiological profiles associated with differing behavioral profiles, however, is not unprecedented. Ersche et al. (2012), for example, found that siblings of individuals suffering from stimulant-dependence had the same underlying neural abnormalities—despite their abstaining from stimulant use. Future studies with a larger sample size are needed to determine if the between group consistency was due to low power or similar neural processing between groups.

Third, while the sample size may have been insufficient to reveal neurobiological differences between groups, it was sensitive to task related differences. Specifically, we found differences between condition activation in the dmPFC and precuneus. Previous studies have found elevated activation of the dmPFC during complex decision-making tasks (Paulus et al., 2002; Pochon et al., 2008; Venkatraman et al., 2009)—consistent with the complexity of making judgements regarding probabilities during the current task. These neural response patterns, however, differ slightly from Abidi et al. (2018) who found elevated activation in the OFC and VS during severe side effect conditions and Miedl et al. (2012) who found a trend toward less pronounced activation in the OFC and VS in gamblers compared to controls during a PD task. Specifically, results showed neural values were attenuated for gamblers during PD tasks (Miedl et al., 2012). Although inconsistent, these results contribute to our overall understanding of the neural correlates of this understudied behavioral process. Additional work is needed to determine the reasons for these discrepancies.

Finally, there were limitations to the study that can be addressed in future research. The first limitation is the small group sizes and large amounts of variability within and between groups that reduced statistical power needed to identify some group level differences. The next limitation is that indifference points from the pre-scan task were entered into the scan computer to equate the task. By equating the tasks, it could be preventing some differences from being identified. It does, however, functionally equate the tasks which reduce differences in task difficulty and differential responding. Equating the tasks sets the expected outcomes equal across groups. This means that observed regional differences are reflective of neurological differences and not tied to task difficulty.

For future studies, neurobiological differences could be investigated as to differences in non-task dependent, resting state activity, outside and inside a gambling environment. Those differences could then be compared to neural activity while gambling in a real-world gambling environment. Additionally, behaviors specific to the gambling environment, such as betting, collecting their winnings or watching their losses being removed could highlight some subtleties that are easily lost in translation to a research study. Further, auditory stimuli need to be investigated to study the impact on neural activity underlying behavioral processes during decision making.

In summary, this study replicated previous findings of PG using PD tasks in an fMRI study, but also highlighted new findings that need to be further investigated. Additionally, these differences need to be evaluated in a larger cohort to gain the necessary statistical power to evaluate some subtleties noted in regional activation differences. Further research is needed to replicate and extend these findings to treatments that may target the mediation of the risky outcome with the reward drive.

## Data Availability Statement

The raw data supporting the conclusions of this article will be made available by the authors, without undue reservation.

## Ethics Statement

The studies involving human participants were reviewed and approved by the University of Kansas Institutional Review Board. The patients/participants provided their written informed consent to participate in this study.

## Author Contributions

TS contributed to the study’s design, conduct, analysis of data, and writing of the final report. JG, VP, and MB contributed substantially to the conduct of the experiment. CD contributed to the design of the experiment and conduct of the experiment. LM contributed to the design of the study, its conduct, data analysis, and writing of the final report. DJ contributed to the conceptualization and design of the study, its conduct, data analysis, and writing of the final report. All authors contributed to the article and approved the submitted version.

## Conflict of Interest

The authors declare that the research was conducted in the absence of any commercial or financial relationships that could be construed as a potential conflict of interest.

## Publisher’s Note

All claims expressed in this article are solely those of the authors and do not necessarily represent those of their affiliated organizations, or those of the publisher, the editors and the reviewers. Any product that may be evaluated in this article, or claim that may be made by its manufacturer, is not guaranteed or endorsed by the publisher.
